# Sexual Orientation-Related Differences in Virtual Spatial Navigation and Spatial Search Strategies

**DOI:** 10.1007/s10508-017-0986-5

**Published:** 2017-04-11

**Authors:** Qazi Rahman, Jonathan Sharp, Meadhbh McVeigh, Man-Ling Ho

**Affiliations:** Department of Psychology, Institute of Psychiatry, King’s College London, Guy’s Hospital Campus, London, SE1 9RT UK

**Keywords:** Sexual orientation, Homosexuality, Spatial memory, Morris water maze, Spatial strategies

## Abstract

Spatial abilities are generally hypothesized to differ between men and women, and people with different sexual orientations. According to the cross-sex shift hypothesis, gay men are hypothesized to perform in the direction of heterosexual women and lesbian women in the direction of heterosexual men on cognitive tests. This study investigated sexual orientation differences in spatial navigation and strategy during a virtual Morris water maze task (VMWM). Forty-four heterosexual men, 43 heterosexual women, 39 gay men, and 34 lesbian/bisexual women (aged 18–54 years) navigated a desktop VMWM and completed measures of intelligence, handedness, and childhood gender nonconformity (CGN). We quantified spatial learning (hidden platform trials), probe trial performance, and cued navigation (visible platform trials). Spatial strategies during hidden and probe trials were classified into visual scanning, landmark use, thigmotaxis/circling, and enfilading. In general, heterosexual men scored better than women and gay men on some spatial learning and probe trial measures and used more visual scan strategies. However, some differences disappeared after controlling for age and estimated IQ (e.g., in visual scanning heterosexual men differed from women but not gay men). Heterosexual women did not differ from lesbian/bisexual women. For both sexes, visual scanning predicted probe trial performance. More feminine CGN scores were associated with lower performance among men and greater performance among women on specific spatial learning or probe trial measures. These results provide mixed evidence for the cross-sex shift hypothesis of sexual orientation-related differences in spatial cognition.

## Introduction

Sex differences in spatial cognition are well documented. Typically, males score higher than females, on average, on spatial tasks involving mental rotation of three-dimensional figures, spatial visualization (such as mental paper folding), disembedding (finding simple figures hidden in more complex forms), spatial perception (determining horizontal and vertical angles), maze navigation, and targeting and intercepting objects (e.g., Kimura, 1999; Voyer, Voyer, & Bryden, 1995). The origins of these differences are likely multifactorial and have been attributed to differences in cerebral lateralization, sociocultural factors (e.g., gender socialization), and the influence of organizational and activational effects of sex hormones (Collaer & Hines, 1995).

Among the largest sex differences are to be found in spatial memory. Spatial memory is an essential cognitive function that allows an organism to locate important objects, places, and conspecifics in either a familiar or new environment. There is an average male advantage on spatial navigation tasks across several formats, including paper-and-pencil tests, computerized mazes, and real-life wayfinding (Choi & Silverman, 1996; Dabbs, Chang, Strong, & Milun, 1998; Galea & Kimura, 1993; Moffat, Hampson, & Hatzipantelis, 1998; Saucier, Green, Leason, MacFadden, & Elias, 2002; Silverman et al., 2000). In addition, there are now several studies reporting a male advantage on virtual reality analogues of classic allocentric or reference memory tests such as the Morris water maze (Astur, Ortiz, & Sutherland, 1998; Astur, Tropp, Sava, Constable, & Markus, 2004; Driscoll, Hamilton, Yeo, Brooks, & Sutherland, 2005; Kober & Neuper, 2011; Parsons et al., 2004; Sandstrom, Kaufman, & Huettel, 1998). Such tasks were developed in part due to evidence for parallel sex differences in rodent models of hippocampal functioning (Jonasson, 2005).

In the Morris water maze, the subject is required to learn to swim directly to a hidden escape platform in a circular pool from each of several release points. Performance is measured as time (latency) to reach the platform over several trials, among other measures (e.g., length taken to reach the platform and the heading error made). Subsequently, the platform is removed during a probe trial and performance is then measured as persistence in searching where the platform had previously been located. In humans, this is often done via a computerized task. The typical finding is that men have faster average search latencies during hidden platform conditions and spend a greater percentage of their time in the platform quadrant compared to women. Therefore, men score higher, on average, than women in spatial learning and the subsequent spatial memory for the target. These sex differences appear to extend to human analogues of other popular rodent mazes, such as the hole-board maze but not the radial arm maze (e.g., Cánovas & Cimadevilla, 2011; Cánovas, Espínola, Iribarne, & Cimadevilla, 2008; Cánovas, Fernández-García, & Cimadevilla, 2011, cf. Astur et al., 2004; Levy, Astur, & Frick, 2005). Thus, these sex differences may be task-specific.

One critical gap in this research concerns the spatial behaviors used to explore spatial environments or “spatial strategies.” It is often argued that men use primarily Euclidean (geometric) cues (such as cardinal directions) to aid navigation while women use landmark or object location information (e.g., Dabbs et al., 1998; Rahman, Andersson, & Govier, 2005; Sandstrom et al., 1998; Saucier et al., 2002). Women’s reliance on landmark cues is consistent with evidence suggesting they encode and recall easily verbalized object identities and locations better than men do (Voyer, Postma, Brake, & Imperato-McGinley, 2007). A difference in spatial strategy raises the question of what is being measured in studies of sexual variation in spatial memory–“ability” or strategy? Since tests such as the Morris water maze are often measured in terms of time taken (e.g., time taken to swim to the platform), the use of a less efficient or “slower” spatial strategy (e.g., using landmarks or swimming close the maze wall, known as thigmotaxis) may result in low performance (McCarthy & Konkle, 2005). Sexual variation in spatial search strategies used to solve the Morris water maze has never been systematically quantified. In fact, many studies using Morris water maze base their results on simple direct measures (e.g., latency and path length) without taking into account other behavioral patterns in the data.

Several candidate search strategies, defined as distinctive motion patterns in the navigation paths, are possible here. Thigmotaxis is a wall-following spatial strategy. Rodent studies indicate that females use thigmotaxis more than males do and that this might be responsible for longer time taken by females to reach the platform (Beiko, Lander, Hampson, Boon, & Cain, 2004; Perrot-Sinal, Kostenuik, Ossenkopp, & Kavaliers, 1996). Thigmotaxis is also associated with greater stress levels, possibly during the early stages of the spatial test (Beiko et al., 2004; Kallai et al., 2007). Other strategies identified by prior research include circling, which is similar to thigmotaxis but involves arc-shaped searches inside the walls of the arena but not close to the wall (Astur et al., 2004; Kallai, Makany, Karadi, & Jacobs, 2005). A visual scan or “direct” strategy involves scanning cues around a fixed position and then taking a clear, directional move (often a straight line) toward the target in the arena (Kallai et al., 2005). A landmark strategy involves moving to a specific location, reorienting, and then moving to the target (Astur et al., 2004). Enfilading involves “zigzagging,” as if moving from one object to another, or involves smaller directional movements (straight lines) followed by rapid zigzag movements (Astur et al., 2004; Gaunet & Thinus-Blanc, 1996; Kallai et al., 2005). These goal-directed responses during navigation may contribute to the formation of a viewer-independent (or allocentric) cognitive map used during water maze-type tasks to promote later spatial recall (O’Keefe & Nadel, 1978).

In terms of neural correlates, the hippocampus plays a strong role in spatial memory processes, especially those tested using Morris water maze-type tasks (O’Keefe & Nadel, 1978). Several human lesion studies report spatial memory impairments following hippocampal damage on allocentric spatial mazes (Astur, Taylor, Mamelak, Philpott, & Sutherland, 2002; Kessels, De Haan, Kappelle, & Postma, 2001; Parslow et al., 2005). Thus, it is possible that sex differences in spatial maze performance are underlain by differences in hippocampal integrity (which, in turn, might be influenced by the multifactorial causes mentioned earlier, including learning and hormonal mechanisms) (Jonasson, 2005). However, the evidence for this is mixed. Several studies suggest no significant sex differences in hippocampal structure and function (Blanch, Brennan, Condon, Santosh, & Hadley, 2004; Good et al., 2001; Janzen & Van Turennout, 2004). In contrast, one large meta-analysis found that males have, on average, larger gray matter volume in the bilateral hippocampi and anterior parahippocampal gyri while females greater volume in the left parahippocampal gyrus (Ruigrok et al., 2014; see also Li et al., 2014).

Growing research shows that sexual orientation is also strongly related to spatial performance. Gay men have lower scores compared to heterosexual men on basic tests of spatial ability, such as mental rotations and judgment of line orientation (but are not significantly different from heterosexual women) (Collaer, Reimers, & Manning, 2007; McCormick & Witelson, 1991; Neave, Menaged, & Weightman, 1999; Rahman & Wilson, 2003; Sanders & Ross-Field, 1986; Sanders & Wright, 1997; Wegesin, 1998). One study has reported no significant sexual orientation difference on mental rotation and spatial perception tests after controlling for measures of general intelligence (Gladue & Bailey, 1995). This study points to the need to control for factors such as IQ. Two studies have reported that gay men have lower performance compared to heterosexual men in spatial navigation (one study using a virtual Morris water maze) and are no different to heterosexual women (Cánovas & Cimadevilla, 2011; Rahman & Koerting, 2008). There are also indications that gay men use more landmark-type strategies during spatial performance although a systematic study of search paths has not yet been conducted (Cánovas & Cimadevilla, 2011; Rahman et al., 2005). Two further studies have found that gay men had greater object location memory compared to heterosexual men (and were no different from heterosexual women) (Hassan & Rahman, 2007; Rahman, Wilson, & Abrahams, 2003). While one study reported that lesbian women were more similar in spatial maze performance to heterosexual men, the bulk of the research on cognitive differences suggests lesbian women are female-typical (Cánovas & Cimadevilla, 2011; cf. Rahman & Koerting, 2008; Rahman et al., 2003). One large, cross-national study has found that some of these cognitive differences were replicable in non-Western groups (Collaer et al., 2007).

In broad terms, this evidence indicates that the cognitive profiles of gay men are “feminized” or are “cross-sex shifted.” That is, where there is a general sex difference in a particular cognitive ability, gay men will perform, on average, in the same direction as heterosexual women. Lesbian women are expected to perform, on average, in the same direction as heterosexual men (or in a more “masculinized” pattern), but the evidence reviewed above does not support the prediction for women. Theoretical accounts for these sexual orientation differences focus on the role of prenatal androgens acting on developing neural circuitry related to sexual orienting mechanisms and associated behavioral correlates (such as cognitive differences) (Collaer & Hines, 1995; Ellis & Ames, 1987). Prenatal sex hormones are predicted to organize both sexual orientation and cognitive ability in sex-atypical directions among gay men and lesbian women. In addition, such hormonal organization is also predicted to influence a constellation of psychological traits that are correlated with sexual orientation, such as childhood and adult gender-typical behavior and interests (Bailey et al., 2016). The pattern of cross-sex shifts in spatial performance among gay men is thus far consistent with this theoretical framework. Further support comes from girls with androgen overexposure in utero (due to congenital adrenal hyperplasia) who show elevated lesbian and bisexual attractions and male-typical performance on the virtual Morris water maze (Mueller et al., 2008; Zucker et al., 1996).

An important developmental factor in sexual orientation-related cognitive differences may be childhood gender nonconformity (CGN). This refers to the level of sex-typed play preferences, behavior, and interests during childhood. Studies have shown that gay men are, on average, more feminine in behavior, feelings, and interests during childhood compared to heterosexual men. Lesbian women are somewhat more masculine, on average, in these respects relative to heterosexual women. The robust relationship between CGN and sexual orientation has been shown in prospective and retrospective studies (Bailey & Zucker, 1995; Steensma, van der Ende, Verhulst, & Cohen-Kettenis, 2013), in cross-cultural research (Cardoso, 2009), and study designs which control for retrospective memory biases (Rieger, Linsenmeier, Gygax, & Bailey, 2008). One study found that CGN was an independent predictor of specific object location memory scores (Hassan & Rahman, 2007). Another study reported an association between CGN and verbal IQ scores among heterosexual men and women but not gay men (Rahman, Bhanot, Emrith-Small, Ghafoor, & Roberts, 2012). These associations were small but appear to support the hypothesis that CGN captures some of the variation in the psychological correlates (e.g., cognition) associated with sexual orientation (Bailey, Dunne, & Martin, 2000). From a theoretical perspective, CGN is thought to be linked to sexual orientation through common mechanisms which may include genetic factors or prenatal sex hormones (e.g., see Bailey et al., 2000). Thus, it is possible that sex-atypical sexual orientation (i.e., homosexuality), spatial cognition, and gender nonconformity are tied together by this common mechanism. Of course, such a mechanism need not be non-social in origin although the existing evidence points in this direction (Bailey et al., 2016).

The aims of the present study were twofold. Firstly, to quantify sexual orientation-related group differences in spatial learning, spatial memory, and use of spatial search strategies during a virtual Morris water maze (VMWM). There are no studies of sexual orientation-related differences in spatial strategy using well-characterized tasks like the VMWM. Further, the VMWM gives several dependent measures than have not been previously studied in relation to sex and sexual orientation (e.g., heading errors). Therefore, this study examined more systematically the range of dependent measures offered by this task. Secondly, this study tested whether CGN is independently associated with any significant VMWM variables. Based on the existing evidence for a cross-sex shift in the spatial performance of gay compared to heterosexual men, we predicted that heterosexual men would outperform heterosexual women, gay men, and lesbian women on spatial learning and spatial memory during the VMWM. Lesbian women were not predicted to differ from heterosexual women based on the past literature. In addition, we predicted that heterosexual men would use a more “direct” spatial strategy (such as visual scanning) during spatial learning compared to heterosexual women, gay men, and lesbian women (who would use more landmark or thigmotaxic strategies). Finally, we predicted that heterosexual men would have significantly more masculine CGN scores than the other groups, consistent with prior findings, and that masculine CGN scores would be independently associated with better spatial performance in men and women.

## Method

### Participants

Using a medium effect size (η_*p*_^2^ = .059) from previous studies of sexual orientation and cognition, a power analysis for an *F*-test was computed (for repeated measures with four groups, between-subjects, with spatial learning and strategies outcome measurements aggregated into 5 blocks). This indicated we would need at least 140 participants in total with statistical power at 90% to detect a medium effect size significant at the 5% level. A total of 160 participants (aged 18–54 years) participated in this study, including 44 heterosexual men, 43 heterosexual women, 39 gay men, and 34 lesbian/bisexual women. They were recruited from the King’s College London student population and through student and community lesbian, gay, and bisexual organizations. Participants were recruited through convenience sampling via electronic and paper-based adverts in student and community outlets.

Sexual orientation was assessed using responses to a sexual identity label item (heterosexual/straight, bisexual, or gay/lesbian) and an item about sexual feelings (defined as attractions toward same or opposite sex) on a 7-point Kinsey-type scale (0 = exclusively heterosexual, 6 = exclusively homosexual). The polychoric correlation between sexual identity and sexual attraction for the whole group was very high (*r* = .98) and the heterosexual and homosexual groups appeared neatly separated on their sexual attraction scores (heterosexual men *M* = .27, SD = .64; heterosexual women *M* = .40, SD = .54; gay men *M* = 5.77, SD = .48; lesbian/bisexual women *M* = 4.88, SD = 1.85, *F*(3, 159) = 491.66, *p* < .001). Note, because women report more bisexuality and have a sexual orientation that is substantially less category-specific compared to men we included bisexual women (responding “bisexual” to identity and 2, 3 or 4 to sexual attractions, *N* = 5) with lesbian women (Bailey et al., 2016).

Age and number of years spent in full-time education since the age of 5 was recorded. Ethnicity was recorded by participants checking one of 18 options according to the Office of National Statistics (ONS) 2011 Census for England Guide on Methodology (http://goo.gl/B2eHtK; its use was required by our ethics committee). For ease, the 18 options were collapsed into five categories labeled White; mixed/multiple ethnic groups; Asian/Asian British; Black/African/Caribbean/Black British; and other ethnic group.

### Measures and Procedure

The study received ethical approval from the King’s College London Psychiatry, Nursing and Midwifery Research Ethics Sub-Committee (reference PNM/12/13-163). All participants gave written informed consent. Participants were tested individually and completed questionnaire measures, followed by a perceived stress scale, then the virtual reality task, and a final perceived stress scale.

#### Perceived Stress

Women tend to report greater spatial anxiety than men, and levels of stress may impair spatial memory performance in a sex-specific manner (Guenzel, Wolf, & Schwabe, 2014; Lawton, 1994; Lawton & Kallai, 2002). Thus, we asked participants to rate their perceived current levels of stress (“Please circle your current level of stress on the following scale”) on a single-item scale ranging from 0 (not at all stressed) to 7 (extremely stressed). This was done immediately before and then after the VMWM.

#### Handedness

This was evaluated using the Edinburgh Handedness Inventory (EHI; Oldfield, 1971), requiring participants to state the degree of preference for the hand used during 10 unimanual tasks. A handedness quotient was calculated by subtracting the score for the left hand from the score for the right hand, dividing by the sum of both, and multiplying by 100, providing an absolute range from −100 (completely left-handed) to +100 (completely right-handed). For this study, we did not specifically recruit participants with a particular type of handedness preference. However, handedness has been previously associated with sexual orientation (Lalumière, Blanchard, & Zucker, 2000). Thus, we wanted to test for potential group differences in EHI scores.

#### Estimated Intelligence

The National Adult Reading Test (NART) provided an estimate of Wechsler Adult Intelligence Scale Full Scale IQ (FSIQ) scores. Participants were required to read out loud 50 short irregular English words, ordered in increasing difficulty. The total numbers of pronunciation errors recorded were converted to predicted Wechsler Adult Intelligence Scale (WAIS) Full Scale IQ scores using the NART manual (Nelson, 1982).

#### Recalled Childhood Gender Nonconformity (CGN)

This 10-item scale asked participants to rate their sex-typed behavior and interests (e.g., play peer preferences, interest in rough-and-tumble play) from as early as they could remember up to 12 years on a 5-point scale (4 items were reversed scored). The items were based on those published by Zucker et al. (2006), but the wording was amended for a British sample (Hassan & Rahman, 2007; Rahman et al., 2012). High average scores reflect feminine behavior and interests.

#### Virtual Morris Water Maze (VMWM) Task

The VMWM used has been described in detail elsewhere (Hamilton, Driscoll, & Sutherland, 2002; Hamilton & Sutherland, 1999). The task is presented on a laptop computer screen, viewed from a first-person perspective (from the viewpoint of the participant and not a “birds eye view”) with the monitor displaying a 45° field of view. The “environment” that the participant sees on the computer screen is a circular pool in the middle of a room with a square floor plan. The viewer’s position was slightly above the surface of the pool. Participants had to move as quickly as possible (using keyboard arrow keys) through the pool toward a hidden platform using distal cues (icons) surrounding the pool. Forward movement was controlled by the up arrow key and rotation to the left and the right controlled by the left and right arrow keys (e.g., if they pressed the right arrow key, the view on the screen would pan to the right). Movement was continuous as long as participants were pressing the arrow keys. Backward navigation or up-down movement was not possible. The platform was positioned in the North-East quadrant of the pool. The icons (or “landmarks”) were a window, a door, a painting, and a bookshelf (one icon per wall). The pool was blue in color and textured, and the arena walls were light gray and not textured.

There were three phases to the task. During a “hidden platform” phase, participants started from four different cardinal locations, four times (randomized) over 20 trials (60 s each) with an ITI of 2 s. If a participant swam over the hidden platform, a text box and tone indicated success. If the participant had not found the platform in the allotted time, the platform was made visible and a text box and tone informed the participant that this was so. Upon finding the platform, the participant was able to look around the room (e.g., at the icons) while upon the platform for 5 s before the maze was reset for the next trial. After this phase, a probe trial was given in which the platform was removed and free search permitted to find the platform for 45 s after which the trial was terminated. No indication was provided that this trial was different from the previous phase. Finally, a control “visible platform” phase required participants to swim to a raised platform for 8 trials.

#### Spatial Learning

From the hidden trials phase, the latency to platform, path length to platform (relative to pool diameter), speed (path length X latency), percentage of time spent in the platform quadrant, and heading error (the angular deviation from a straight trajectory to the platform from the starting position) were recorded. These were binned into 5 blocks: a first block of 3 trials (the first trial was designated as a practice and so we excluded it) and then four blocks of 4 trials each.

#### Spatial Memory

This was measured from the probe trial and included path length to reach platform quadrant, percentage of time in platform quadrant, and heading error.

#### Cued Navigation

This was measured via latency, path length, speed (path length X latency), and heading error during visible trials (binned into 2 blocks of 4 trials each).

#### Spatial Strategies

“Swim” paths for each participant during each hidden trial and the probe trial were recorded by the software as a bitmap picture. These visual data were categorized into one of four spatial strategies (visual scan, landmark, thigmotaxis/circling, and enfilading) by visual inspection using the category descriptors of Astur et al. (2004) and Kallai et al. (2005). Three judges blind to sex and sexual orientation categorized each swim path by consensus into one of the four categories according to the descriptors. Where the raters could not agree, the swim path was excluded (295/9.22%).

*Visual Scan* is a strategy where participants rotate around a fixed position and swim in a direct line toward the platform. Astur et al. (2004) also refer to this as a “direct strategy” and argue that it involves the greatest amount of spatial processing compared to other strategies. The strategy appears as a small spot on the image of the path and then a direct, often straight, line toward the platform. A *Landmark* strategy describes a path where the participant swims directly toward one location in the pool, rotates clearly, and then swims toward the platform. This involves a rotation or pivot at one point only along the path. *Thigmotaxis/Circling* is where participants follow a circular path close to the wall. Kallai et al. (2005) also described a strategy named circling where participants swim in a circle, or in large arcs, at a certain distance away from the wall. However, we were unable to distinguish clearly between thigmotaxis and circling here. For example, participants showed a pattern where they would move very close along the wall (as if to “touch” it) for some of the path and then moved away from it for the remainder (or a mix of touching and then moving away from the wall throughout the path). Thus, we included both strategies in the same category. *Enfilading* is defined as small, angular directional changes interspersed with straight lines of movement toward the platform. On the image of the path, this often looks like a “back-and-forth” or “zigzag” type of motion (Astur et al., 2004; Kallai et al., 2005). Scoring for this part of the study included the number of times each of the four strategies was used during the hidden platform latency trials (binned into blocks as described above) and the probe trial.

### Statistical Analysis

Group differences in demographic (except ethnicity), handedness, IQ, and CGN variables were analyzed using univariate ANOVA. For post hoc analysis, LSD tests were used comparing heterosexual men with heterosexual women; heterosexual men with gay men; and heterosexual women with lesbian/bisexual women. VMWM performance (except for the probe trial) was analyzed using repeated measures ANOVA with group as the between-subjects factor and trial block as the within-subjects factor (using Greenhouse-Geisser correction if the assumption of sphericity was not met).^1^ Univariate ANOVA was used to analyze the probe trial. Post hoc LSD tests were used to unpack significant group differences on the estimated means due to the directional nature of our hypotheses. Significant group differences were followed up with repeated measures analysis of covariance (ANCOVA) to control for age and estimated IQ. This was done in order to show any change in the pattern of results before and after controlling for important covariates. Post hoc LSD tests were also used on the estimated means (as these are adjusted for covariates in the model). Effect sizes are reported as η_*p*_^2^ or Cohen’s *d* where appropriate (Cohen, 1988). Here, η_*p*_^2^ = .01 is regarded as a small effect, η_*p*_^2^ = .05 a medium effect, and η_*p*_^2^ ≥ .13 a large effect. For Cohen’s *d*, *d* = .20 is regarded as a small effect, *d* = .50 a medium effect, and *d* ≥ .80 a large effect.

To investigate the independent contribution of CGN to VMWM performance, multiple regression analyses were conducted separately for men and women, one each for VWMW outcomes with significant group differences. In each regression, predictor variables were sexual orientation (dummy coded with heterosexual men or heterosexual women as the reference group), CGN, age, estimated IQ, and the spatial strategy used during the hidden or probe trial (as we expect spatial strategies to be a predictor of spatial performance: Kallai et al., 2005).

## Results

### Participant Characteristics

There were no significant group differences in age, *F*(3, 155) = 2.51, *p* = .06, years spent in education, *F*(3, 151) = .54, *p* = .65, handedness scores, *F*(3, 154) = 1.86, *p* = .14, estimated IQ, *F*(3, 144) = 1.84, *p* = .14, perceived stress before the VMWM, *F*(3, 150) = 1.25, *p* = .29, and perceived stress after the VMWM, *F*(3, 150) = 1.30, *p* = .28 (Table 1). The CGN scale had high internal consistency (Cronbach’s *α* = .88). There was an expected significant group difference in CGN scores, *F*(3, 152) = 54.31, *p* < .001. Heterosexual men were significantly more masculine scoring than heterosexual women (mean difference = −1.51, SE = .11, *p* < .001, *d* = 3.45) and gay men (mean difference = −.60, SE = .12, *p* < .001, *d* = 1.22). Heterosexual women were significantly more feminine than lesbian/bisexual women (mean difference = .74, SE = .12, *p* < .001, *d* = 1.26).

**Table 1:** Mean scores (and SD) for sample demographic and sexual orientation characteristics by group

Variable	Heterosexual men (*N* = 44)		Heterosexual women (*N* = 43)		Gay men (*N* = 39)		Lesbian/bisexual women (*N* = 34)	
	*M*	SD	*M*	SD	*M*	SD	*M*	SD
Age (years)	26.73	10.76	26.03	8.55	31.39	11.12	26.88	7.45
Years in education	15.95	2.17	16.87	3.28	16.28	4.50	16.20	3.19
Edinburgh Handedness Inventory^a^	58.93	55.69	78.20	33.85	55.53	67.90	72.16	30.59
Estimated IQ	111.25	4.88	110.81	4.72	113.51	6.08	111.68	5.94
Sexual attractions^b^	.27	.62	.40	.54	5.77	.48	4.88	1.45
Childhood gender nonconformity^c^	1.94	.39	3.45	.48	2.54	.58	2.71	.69
Perceived stress before VMWM	2.37	1.77	2.63	1.72	2.38	1.80	3.08	1.83
Perceived stress after VMWM	2.12	1.45	2.43	1.88	2.48	1.62	2.91	1.88

Estimated IQ = estimated from the NART (National Adult Reading Test)

^a^Absolute range −100 to 100

^b^Absolute range 0–6

^c^Absolute range 1–5

We collapsed ethnicity into “White” and “non-White” categories. Ninety-seven of the 160 participants were White, and the remaining were non-White. The groups did not differ in ethnicity, *χ* ^2^(3) = 5.69, *p* = .12. We controlled for age and estimated IQ because these are well-known covariates of cognitive ability (Lezak, 1995).

### Spatial Learning

In order to reduce within-group variability, we averaged performance for trial blocks 2–3 and trial blocks 4–5 for the analyses below (Table 2).

**Table 2:** Unadjusted mean scores (and SD) for virtual Morris water maze performance outcomes by group

Variable	Heterosexual men (*N* = 44)		Heterosexual women (*N* = 43)		Gay men (*N* = 39)		Lesbian/bisexual women (*N* = 34)	
	*M*	SD	*M*	SD	*M*	SD	*M*	SD
Spatial learning (hidden trials)								
Latency to platform block 1 (s)	27.68	15.01	37.81	16.48	38.26	17.60	35.17	17.11
Latency to platform blocks 2 and 3 (s)	18.66	12.30	29.62	12.62	30.68	16.01	29.18	15.72
Latency to platform blocks 4 and 5 (s)	16.39	12.75	24.18	12.34	26.27	16.52	23.17	14.00
Path length to platform block 1	2.38	1.45	2.59	1.53	2.48	1.38	2.63	1.57
Path length to platform blocks 2 and 3	1.62	1.32	2.27	1.10	2.03	1.22	2.12	1.40
Path length to platform blocks 4 and 5	1.33	1.29	1.84	1.28	1.76	1.37	1.66	1.18
Speed block 1	.07	.01	.06	.02	.06	.02	.06	.02
Speed blocks 2 and 3	.06	.02	.07	.02	.06	.02	.07	.02
Speed blocks 4 and 5	.07	.02	.07	.02	.06	.02	.07	.06
Time in platform quadrant block 1 (%)	39.62	18.61	31.80	13.06	32.82	18.14	36.30	17.22
Time in platform quadrant blocks 2 and 3 (%)	50.67	17.81	38.66	15.82	41.54	17.61	43.73	15.81
Time in platform quadrant blocks 4 and 5 (%)	53.59	16.26	45.63	16.90	46.41	19.24	48.59	17.73
Heading error block 1 (°)	37.81	20.51	41.64	16.59	40.38	20.48	44.73	19.68
Heading error blocks 2 and 3 (°)	28.75	19.60	39.24	17.43	38.99	17.64	36.05	20.01
Heading error blocks 4 and 5 (°)	21.93	17.20	31.91	17.99	32.89	22.39	30.78	19.82
Spatial memory (probe trial)								
Path length to platform	4.24	.87	3.72	1.09	3.22	1.16	3.53	1.15
Time in platform quadrant (%)	57.10	20.30	39.25	23.02	48.87	28.78	48.43	25.61
Heading error (°)	24.48	28.12	32.97	26.49	31.22	29.10	29.13	28.66
Cued navigation (visible trials)								
Latency to platform block 1 (s)	6.81	.98	7.43	.89	8.21	1.80	7.58	1.00
Latency to platform block 2 (s)	6.21	.81	7.19	.87	7.31	1.15	6.95	.79
Path length to platform block 1	.40	.01	.41	.04	.44	.13	.41	.04
Path length to platform block 2	.43	.11	.41	.08	.40	.03	.40	.03
Speed block 1	.06	.00	.05	.00	.05	.01	.05	.00
Speed block 2	.07	.01	.06	.00	.05	.00	.06	.00
Heading error block 1 (°)	3.41	3.00	4.25	6.81	5.57	11.49	4.50	11.32
Heading error block 2 (°)	6.07	10.03	3.70	8.25	4.64	9.84	3.26	9.23

#### Latency

There was a significant main effect of group, *F*(3, 156) = 6.24, *p* < .001, η_*p*_^2^ = .10 (Table 2). Post hoc analysis showed significantly shorter latencies recorded by heterosexual men compared to heterosexual women (mean difference = −9.62, SE = 2.74, *p* = .001, *d* = .76) and gay men (mean difference = −10.82, SE = 2.82, *p* < .001, *d* = .85). Heterosexual women did not differ significantly from lesbian/bisexual women (mean difference = 1.36, SE = 2.94, *p* = .64, *d* = .10). After controlling for age and IQ, heterosexual men still recorded significantly shorter latencies than heterosexual women (mean difference = −10.47, SE = 2.88, *p* < .001, *d* = .83) and gay men (mean difference = −8.38, SE = 3.06, *p* = .007, *d* = .65) while heterosexual and lesbian/bisexual women did not differ (mean difference = 2.38, SE = 3.03, *p* = .43, *d* = .19).

#### Path Length

There was no significant main effect of group, *F*(3, 156) = 1.38, *p* = .24, ηp2 = .02.

#### Speed

There was a significant main effect of group, *F*(3, 156) = 3.22, *p* = .02, η_*p*_^2^ = .06. Heterosexual men were faster than gay men (mean difference = .012, SE = .004, *p* = .002, *d* = .68) but not heterosexual women (mean difference = .007, SE = .004, *p* = .08, *d* = .36). Heterosexual and lesbian/bisexual women did not differ (mean difference = .001, SE = .004, *p* = .88, *d* = .05). However, these differences disappeared after controlling for age and IQ (all *p*s > .05).

#### Time in Platform Quadrant

There was a significant main effect of group, *F*(3, 156) = 3.73, *p* = .01, η_*p*_^2^ = .07. Heterosexual men spent more time in the platform quadrant than heterosexual women (mean difference = 9.26, SE = 2.96, *p* = .002, *d* = .68) and gay men (mean difference = 7.70, SE = 3.03, *p* = .01, *d* = .57). Heterosexual and lesbian/bisexual women did not differ from each other (mean difference = −4.17, SE = 3.16, *p* = .19, *d* = .30). After controlling for age and estimated IQ, heterosexual men remained significantly different from heterosexual women (mean difference = 9.21, SE = 3.16, *p* = .004, *d* = .66) but no longer to gay men (mean difference = 6.46, SE = 3.36, *p* = .05, *d* = .46). Heterosexual and lesbian/bisexual women did not differ (mean difference = −4.03, SE = 3.34, *p* = .22, *d* = .29).

#### Heading Error

There was a significant main effect of group, *F*(3, 156) = 2.74, *p* = .04, η_*p*_^2^ = .05. Heterosexual men had a lower heading error than heterosexual women (mean difference = −8.10, SE = 3.35, *p* = .01, *d* = .53) and gay men (mean difference = −7.92, SE = 3.43, *p* = .02, *d* = .51). Heterosexual and lesbian/bisexual women did not differ significantly (mean difference = .41, SE = 3.58, *p* = .90, *d* = .02). However, these differences disappeared after controlling for age and IQ (all *p*s > .05).

### Spatial Memory

#### Path Length

There was a significant main effect of group on path length, *F*(3, 156) = 6.63, *p* < .001, η_*p*_^2^ = .11 (Table 2). Heterosexual men travelled further than heterosexual women (mean difference = .52, SE = .23, *p* = .02, *d* = .50) and gay men (mean difference = 1.02, SE = .24, *p* = .001, *d* = .96). Heterosexual and lesbian/bisexual women did not differ significantly from each other (mean difference = .19, SE = .24, *p* = .43, *d* = .18). This remained after controlling for the covariates. Heterosexual men were still significantly different to heterosexual women (mean difference = .61, SE = .23, *p* = .01, *d* = .59) and gay men (mean difference = .84, SE = .25, *p* = .001, *d* = .81). Heterosexual and lesbian/bisexual women did not differ (mean difference = .05, SE = .25, *p* = .83, *d* = .06).

#### Time in Platform Quadrant

There was a significant main effect of group, *F*(3, 156) = 3.87, *p* = .01, η_*p*_^2^ = .07. Heterosexual men spent significantly more time in the platform quadrant than heterosexual women did (mean difference = 17.84, SE = 5.24, *p* = .001, *d* = .74). Heterosexual men did not differ significantly from gay men (mean difference = 8.22, SE = 5.37, *p* = .13, *d* = .34). Heterosexual and lesbian/bisexual women did not differ significantly (mean difference = −9.18, SE = 5.60, *p* = .10, *d* = .38). This remained after controlling for the covariates. Heterosexual men were still significantly different to heterosexual women (mean difference = 16.34, SE = 5.64, *p* = .004, *d* = .66) and no different to gay men (mean difference = 4.73, SE = 5.99, *p* = .43, *d* = .19). Heterosexual women did not differ from lesbian/bisexual women did not differ (mean difference = −8.83, SE = 5.94, *p* = .14, *d* = .36).

#### Heading Error

There was no effect of group on heading error, *F*(3, 156) = .74, *p* = .53, η_*p*_^2^ = .01.

### Cued Navigation

#### Latency

There was a significant main effect of group, *F*(3, 145) = 10.85, *p* < .001, η_*p*_^2^ = .18 (Table 2). Post hoc analysis showed significantly shorter latencies recorded by heterosexual men compared to heterosexual women (mean difference = −.80, SE = .22, *p* < .001, *d* = .82) and gay men (mean difference = −1.25, SE = .23, *p* < .001, *d* = 1.30). Heterosexual women did not differ significantly from lesbian/bisexual women (mean difference = .04, SE = .24, *p* = .86, *d* = .04). After controlling for age and estimated IQ, heterosexual men still recorded significantly shorter latencies compared to heterosexual women (mean difference = −.84, SE = .22, *p* < .001, *d* = .92) and gay men (mean difference = −1.02, SE = .24, *p* = .007, *d* = 1.10) while heterosexual and lesbian/bisexual women did not differ (mean difference = .14, SE = .24, *p* = .56, *d* = .15).

#### Path Length

There was no significant main effect of group, *F*(3, 156) = .42, *p* = .74, η_*p*_^2^ = .008.

#### Speed

There was a significant main effect of group, *F*(3, 156) = 13.97, *p* < .001, η_*p*_^2^ = .21. Heterosexual men were faster than heterosexual women (mean difference = .01, SE = .002, *p* < .001, *d* = 1.38) and gay men (mean difference = .01, SE = .002, *p* < .001, *d* = 1.88). Heterosexual and lesbian/bisexual women did not differ (mean difference < .001, SE = .002, *p* = .96, *d* = .11). After controlling for age and IQ, heterosexual men were still faster than heterosexual women (mean difference = .01, SE = .002, *p* < .001, *d* = 1.63) and gay men (mean difference = .01, SE = .002, *p* < .001, *d* = 1.63) while heterosexual and lesbian/bisexual women did not differ (mean difference = −.001, SE = .002, *p* = .54, *d* = .23).

#### Heading Error

There was no significant main effect of group, *F*(3, 156) = .21, *p* = .89, η_*p*_^2^ = .004.

### Spatial Strategies During Spatial Learning (Hidden Trials)

**Fig. 1:**
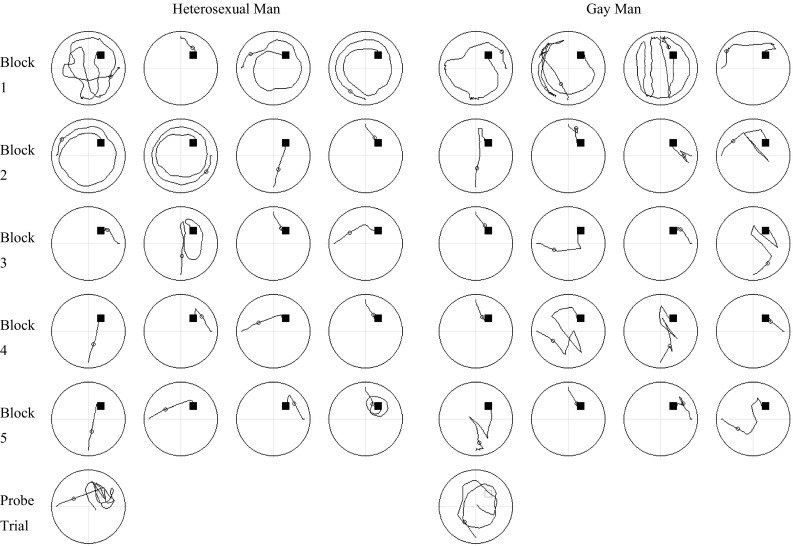
Swim paths for each of the 20 hidden platform (spatial learning) trials and the probe trial for one heterosexual man and one gay man who performed at the median level for their respective groups. Paths for individual trials are ordered from *left to right* within each trial block

**Fig. 2:**
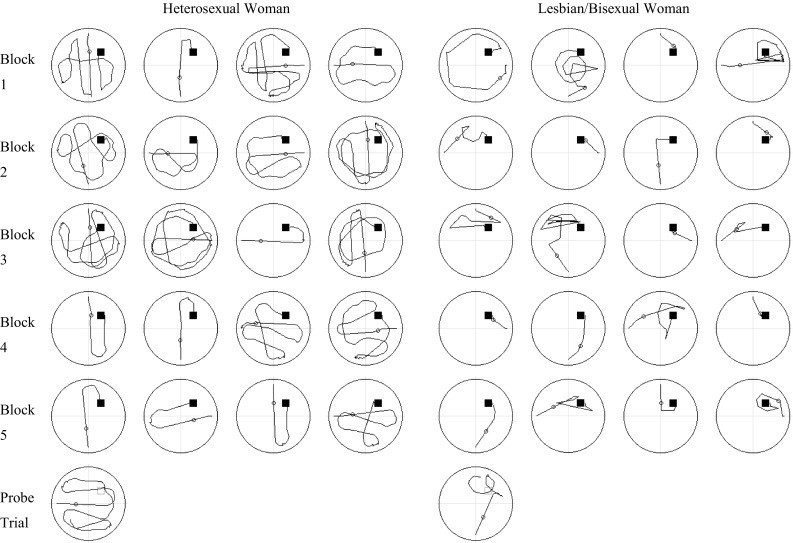
Swim paths for each of the 20 hidden platform (spatial learning) trials and the probe trial for one heterosexual woman and one lesbian/bisexual woman who performed at the median level for their respective groups. Paths for individual trials are ordered from *left to right* within each trial block

We averaged performance for trial blocks 2–3 and trial blocks 4–5 (Table 3). Note that visual scan was used more than the other strategies across all hidden trials, excluding Trial 1 (visual scan = 1126 times, landmark = 372 times, thigmotaxis/circling = 716, and enfilading used 285 times). Figures 1 and 2 show the paths taken during the hidden trials and probe trial for a representative participant from each group.

**Table 3:** Unadjusted mean scores (and SD) and frequencies (for the probe trial) for spatial strategies used by group

Variable	Heterosexual men (*N* = 44)		Heterosexual women (*N* = 43)		Gay men (*N* = 39)		Lesbian/bisexual women (*N* = 34)	
	*M*	SD	*M*	SD	*M*	SD	*M*	SD
Spatial learning (hidden trials)								
Visual scan block 1	.93	.10	.44	.59	.49	.82	.59	.70
Visual scan blocks 2 and 3	3.73	2.74	2.19	2.16	2.36	2.32	2.88	2.61
Visual scan blocks 4 and 5	4.45	2.82	3.28	2.65	3.36	2.89	3.26	2.59
Landmark block 1	.23	.64	.16	.37	.21	.52	.29	.46
Landmark blocks 2 and 3	1.00	1.06	.98	1.08	1.13	1.34	.59	.89
Landmark blocks 4 and 5	1.05	.94	1.16	1.40	1.33	1.46	1.14	1.33
Thigmotaxis/circling block 1	1.00	.94	1.05	1.11	1.08	1.09	1.00	.98
Thigmotaxis/circling blocks 2 and 3	1.55	2.34	2.21	2.35	2.26	2.40	1.97	2.40
Thigmotaxis/circling blocks 4 and 5	1.00	1.99	1.39	2.12	1.92	2.74	1.59	2.43
Enfilading block 1	.55	.82	.93	1.06	.69	.95	.74	.93
Enfilading blocks 2 and 3	1.02	1.75	1.86	2.11	1.36	1.98	1.62	2.36
Enfilading blocks 4 and 5	1.05	1.95	1.42	2.10	.85	1.89	1.53	2.22
Spatial memory (probe trial)								
Visual scan (count)	30		9		15		13	
Landmark (count)	6		8		3		5	
Thigmotaxis/circling (count)	5		10		11		7	
Enfilading (count)	3		11		7		7	
Visual scan versus other strategy	.30	.46	.76	.43	.58	.50	.59	.50

“Visual scan versus other strategy” is a dichotomized variable (0 = visual scan, 1 = any other strategy)

#### Visual Scan

For visual scan, there was a significant main effect of group, *F*(3, 156) = 3.35, *p* = .02, η_*p*_^2^ = .06 (Table 3). Heterosexual men used visual scan significantly more compared to heterosexual women (mean difference = 1.06, SE = .37, *p* = .005, *d* = .63) and gay men (mean difference = .97, SE = .38, *p* = .012, *d* = .58). Heterosexual women did not differ significantly from lesbian/bisexual women (mean difference = −.28, SE = .40, *p* = .49, *d* = .17). After controlling for age and estimated IQ, heterosexual men still used visual scan more than heterosexual women did (mean difference = 1.11, SE = .39, *p* = .005, *d* = .65) but were no longer significantly different to gay men (mean difference = .79, SE = .42, *p* = .06, *d* = .46). Heterosexual and lesbian/bisexual women still did not differ from each other (mean difference = −.28, SE = .41, *p* = .49, *d* = .17).

#### Landmark

There was no significant main effect of group, *F*(3, 156) = .55, *p* = .65, η_*p*_^2^ = .01.

#### Thigmotaxis/Circling

There was no significant main effect of group, *F*(3, 156) = .89, *p* = .45, η_*p*_^2^ = .01.

#### Enfilading

There was no significant main effect of group, *F*(3, 156) = 1.34, *p* = .26, η_*p*_^2^ = .02.

### Spatial Strategies During Spatial Memory (Probe Trial)

The frequencies (counts) for each strategy used by the groups are shown in Table 3. Given the dominance of visual scan as a strategy during spatial learning and the probe trial, we dichotomized probe trial strategy into visual scan versus any other strategy. Univariate ANOVA revealed a significant main effect of group, *F*(3, 146) = 7.00, *p* < .001, η_*p*_^2^ = .13 (Table 3). Heterosexual men used more visual scan during the probe trial compared to any other strategy than heterosexual women (mean difference = −.47, SE = .10, *p* < .001, *d* = 1.00) and gay men (mean difference = −.29, SE = .10, *p* = .007, *d* = .62). Heterosexual and lesbian/bisexual women did not differ significantly (mean difference = .17, SE = .11, *p* = .13, *d* = .36). After controlling for age and IQ, heterosexual men were still significantly different from heterosexual women (mean difference = −.41, SE = .11, *p* < .001, *d* = .86) but no longer from gay men (mean difference = −.18, SE = .12, *p* = .13, *d* = .37). Heterosexual and lesbian/bisexual women did not differ (mean difference = .17, SE = .12, *p* = .17, *d* = .36).

### Spatial Strategies During Cued Navigation (Visible Trials)

It was not meaningful to analyze group differences in spatial strategies during visible trials because visual scan was used almost exclusively during this condition (1248/1280 data points or 98%).

### CGN and Spatial Performance

Regression models were conducted separately on the spatial learning (hidden trials) and probe trial outcomes that continued to yield significant results after the application of the ANCOVA models. These were hidden trial latency, hidden trial time in platform quadrant, probe trial path length, and probe trial time in platform quadrant. Hidden trial-dependent variables used here were computed by averaging performance across all blocks. Predictor variables were group (dummy coded with either heterosexual men or heterosexual women as the reference), CGN, age, estimated IQ, visual scan, landmark, thigmotaxis/circling, and enfilading strategies (for the probe trial model, the variable “visual scan versus other strategy” was included instead of the four strategies used for hidden trials).

#### Men

Initial regression models for hidden trial latency and time in platform quadrant revealed inflated *R* values (*R* = .84, *R* ^2^ = .70, adjusted *R* ^2^ = .66, and *R* = .92, *R* ^2^ = .86, adjusted *R* ^2^ = .84, respectively). This was due to the high correlation between visual scan and the dependent variables (*r* = −.77 and *r* = .89, respectively) and multicollinearity-affected visual scan (tolerance = .10, VIF = 9.97, and tolerance = .10, VIF = 9.98, respectively) and thigmotaxis/circling variables (tolerance = .09, VIF = 10.07, and tolerance = .09, VIF = 10.07, respectively). Thus, visual scan and thigmotaxis/circling were removed from the models presented here.

The model for hidden trial latency was significant, *F*(6, 76) = 7.78, *p* < .001 (*R* = .63, *R* ^2^ = .40, adjusted *R* ^2^ = .35). Gay men had longer latencies than the heterosexual male reference group and more feminine CGN scores were associated with longer latencies as was the use of enfilading. No other predictors were significant (Table 4). The model for hidden trial time in platform quadrant was also significant, *F*(6, 76) = 5.10, *p* < .001 (*R* = .55, *R* ^2^ = .31, adjusted *R* ^2^ = .25). More feminine CGN scores and use of enfilading were associated with less time in the platform quadrant, while landmark strategy usage was associated with more time in the platform quadrant. No other predictors were significant.

**Table 4:** Regression models predicting spatial performance-dependent variables for men

	Hidden trials latencies			Hidden trials time in platform quadrant			Probe trial path length			Probe trial time in platform quadrant		
	*B*	SE *B*	*β*	*B*	SE *B*	*β*	*B*	SE *B*	*β*	*B*	SE *B*	*β*
Gay men^a^	6.86	3.28	.24*	−3.34	3.72	−.11	−.77	.23	−.35**	2.06	4.81	.04
Age	−.01	.15	−.01	.09	.17	.06	−.03	.01	−.28**	.28	.24	.11
Estimated IQ	.35	.25	.13	−.15	.28	−.05	.01	.01	.05	−.61	.39	−.14
Childhood gender nonconformity	6.38	2.70	.25*	−8.39	3.06	−.32**	−.43	.21	−.22*	4.16	4.28	.10
Landmark	−.62	.63	−.09	2.10	.72	.30**	–	–	–	–	–	–
Enfilading	1.47	.37	.38**	−.97	.42	−.23*	–	–	–	–	–	–
Visual scan versus other strategy	–	–		–	–	–	1.04	.21	.47**	−35.56	4.36	−.76**

Estimated IQ = estimated from the NART (National Adult Reading Test)

* *p* < .05; ** *p* < .01

^a^Dummy coded with heterosexual men as the reference group

The model for probe trial path length was significant, *F*(5, 73) = 11.24, *p* < .001 (*R* = .67, *R* ^2^ = .43, adjusted *R* ^2^ = .41). Group (being a gay man), age (being older), and CGN (more feminine scores) were negatively associated with path length, while probe strategy was positively associated with it. Estimated IQ was not a significant predictor. The model for probe trial time in platform quadrant was significant, *F*(5, 73) = 13.59, *p* < .001 (*R* = .70, *R* ^2^ = .50, adjusted *R* ^2^ = .46). Only probe strategy was negatively associated with time spent in the platform quadrant.

#### Women

As with men, initial models for hidden trial latency and time in platform quadrant revealed inflated *R* values (*R* = .87, *R* ^2^ = .76, adjusted *R* ^2^ = .73, and *R* = .89, *R* ^2^ = .80, adjusted *R* ^2^ = .78, respectively). Again, the correlation between visual scan, latencies, and time in platform quadrant was high (*r* = −.79 and *r* = .82, respectively) and multicollinearity affected visual scan (tolerance = .10, VIF = 9.78, and tolerance = .10, VIF = 9.78, respectively), thigmotaxis/circling (tolerance = .10, VIF = 9.92, and tolerance = .10, VIF = 9.92, respectively), and enfilading variables (tolerance = .10, VIF = 9.49, and tolerance = .10, VIF = 9.49, respectively). Thus, visual scan, thigmotaxis/circling, and enfilading were removed as predictors).

The regression model for hidden trial latency in women was significant, *F*(5, 69) = 7.78, *p* = .009 (*R* = .45, *R* ^2^ = .20, adjusted *R* ^2^ = .14). Age was positively associated and landmark strategy negatively associated with latencies. No other predictors were significant (Table 5). For hidden trial time spent in the platform quadrant, the model was also significant, *F*(5, 69) = 2.49, *p* = .04 (*R* = .40, *R* ^2^ = .16, adjusted *R* ^2^ = .10). Group (being lesbian/bisexual), CGN (more feminine scores), and landmark strategy usage was positively associated with time spent in the platform quadrant. No other predictors were significant. The model for probe trial path length was significant, *F*(5, 62) = 6.37, *p* < .001 (*R* = .59, *R* ^2^ = .35, adjusted *R* ^2^ = .30). Probe strategy usage was positively associated with path length. There were no other significant predictors. Finally, the model for probe trial time in platform quadrant was significant, *F*(5, 62) = 6.64, *p* < .001 (*R* = .60, *R* ^2^ = .36, adjusted *R* ^2^ = .31). Once more, probe strategy usage was the only significant, and negatively associated, predictor.

**Table 5:** Regression models predicting spatial performance-dependent variables for women

	Hidden trials latencies			Hidden trials time in platform quadrant			Probe trial path length			Probe trial time in platform quadrant		
	*B*	SE *B*	*β*	*B*	SE *B*	*β*	*B*	SE *B*	*β*	*B*	SE *B*	*β*
Lesbian/bisexual women^a^	−3.99	3.28	−.16	9.01	3.78	.33*	.06	.29	.02	12.48	6.58	.24
Age	.71	.22	.40**	−.42	.26	−.21	−.03	.02	−.23	−.52	.45	−.14
Estimated IQ	.11	.28	.05	.10	.33	.04	−.01	.02	−.05	.05	.57	.01
Childhood gender nonconformity	−1.27	2.31	−.07	5.56	2.66	.28*	−.13	.21	−.08	7.14	4.71	.19
Landmark	−1.26	.63	−.22*	1.62	.72	.25*	–	–	–	–	–	–
Visual scan versus other strategy	–	–		–	–	–	1.38	.26	.57**	−29.79	5.94	−.54**

Estimated IQ = from the NART (National Adult Reading Test)

* *p* < .05; ** *p* < .01

^a^Dummy coded with heterosexual women as the reference group

## Discussion

We assessed spatial navigation and spatial strategy outcomes during a virtual reality Morris water maze in heterosexual and homosexual men and women. In general, our predictions were supported by the results from the unadjusted ANOVA models. Heterosexual men had significantly faster search latencies, spent more “dwelling” time in the platform quadrant, and had smaller heading errors compared to heterosexual women and gay men during spatial learning. During spatial memory (assessed via the probe trial), heterosexual men navigated further than heterosexual women and gay men but only differed significant from heterosexual women in time spent in the platform quadrant. Heterosexual men also used significantly more visual scanning during hidden and probe trials than heterosexual women and gay men. Heterosexual women did not differ significantly from lesbian/bisexual women as expected. The pattern of effect sizes for these group differences was in the medium range.

However, in the adjusted models, heterosexual men only had faster search latencies during spatial learning and longer path lengths on the probe than heterosexual women and gay men. On the remaining spatial outcomes which showed significant group differences in the unadjusted models, heterosexual men were different only from heterosexual women (except heading error where the between-subjects effect was no longer significant). We note that for time spent in the platform quadrant and use of visual scanning during spatial learning, the difference between heterosexual and gay men just dropped below traditional significance levels and the effect sizes remained modest across models. The addition of age and estimated IQ as covariates reduced some of the effects, suggesting that the influence of these covariates on spatial ability depends somewhat on sexual orientation group membership (e.g., we have previously found that gay men to score slightly higher on the NART in community samples: Rahman et al., 2012). Alternatively, the sample may have been underpowered to detect group differences with the addition of covariates (although the trends were still in the predicted directions judging by the pattern of changes in the effect sizes from unadjusted to adjusted models).

We also found that heterosexual men had significantly shorter search latencies on the cued navigation (visible platform trials) compared to heterosexual women and gay men in both adjusted and unadjusted models. This appears to be due to differences in speed on visible platform trials (one of the largest effect sizes we found here), whereas the group differences between heterosexual and gay men in speed was not significant for hidden trials in the adjusted models. This indicates, tentatively, that heterosexual men have better search latencies during spatial learning than the other groups for reasons other than simple speed.

Inspection of Table 2 shows clearly that heterosexual men started faster than the other groups and maintained this advantage throughout spatial learning trials. This indicates that heterosexual men may be using unique search strategies from the first trial onwards. Indeed, this was borne out by the analysis of spatial strategies. Table 3 shows clearly that heterosexual men used more visual scanning from the first block of hidden trials and maintained the use of this strategy throughout the blocks. Heterosexual men also used more visual scanning during the probe trial than any other strategy compared to the other groups. Thus, on both measures of spatial learning and spatial memory, heterosexual men appear to adopt visual scanning during VWMW type tasks. Heterosexual women, gay men, and lesbian/bisexual women on the other hand tended to use a mixture of non-visual scanning strategies during spatial learning and spatial memory rather than any one specific type of alternative strategy (e.g., thigmotaxis; Beiko et al., 2004; Perrot-Sinal et al., 1996).

The present data were consistent with previous research showing that males perform better than females, on average, on MWM tasks as well on more simple maze-learning and wayfinding tests (e.g., Astur et al., 1998, 2004; Driscoll et al., 2005; Moffat et al., 1998; Saucier et al., 2002). The results were consistent with sex differences found in rodent models of place learning and memory (Jonasson, 2005). The findings also support growing evidence that gay men score lower than heterosexual men on spatial navigation tests as well as tests of basic spatial ability (e.g., Cánovas & Cimadevilla, 2011; Collaer et al., 2007; McCormick & Witelson, 1991; Neave et al., 1999; Rahman & Koerting, 2008; Rahman & Wilson, 2003).

While the results do not directly support the suggestion that both women and gay men use more landmark-type strategies during spatial performance, they do indicate that these groups use a mix of spatial strategies that are alternatives to a direct, visual scanning approach (cf. Dabbs et al., 1998; Rahman et al., 2005; Sandstrom et al., 1998; Saucier et al., 2002). The data support the notion that a less efficient strategy might account, in part, for female-typical performance and indicates that researchers should better quantify other, complex behavioral patterns in spatial memory data (McCarthy & Konkle, 2005). The medium effect sizes reported for the male comparisons were smaller than previous studies and, in general terms, support the notion that the strength of sexual orientation differences in spatial performance is task-specific.

A secondary goal of this study was to test the hypothesis that CGN could independently predict some of the variation in spatial performance outcome measures (in addition to group membership, spatial strategies, age, and estimated IQ). Caution should be exercised in interpreting the results from the regression models due to the small sample sizes (due to collapsing by sex) and the restricted number of spatial outcomes (we used only those showing significant differences in the adjusted models). Among men, CGN predicted search latency during hidden trials, time spent in the platform quadrant during hidden trials, and probe trial path length in the expected directions (more feminine CGN scores were associated with lower performance). This supports previous studies suggesting that CGN is sensitive to within-group variation in cognitive function associated with sexual orientation (Hassan & Rahman, 2007; Rahman et al., 2012). But it is unclear what the significance of the association between CGN and spatial performance is. One other study indicated deficits in spatial subtests of the Wechsler Preschool and Primary Scale of Intelligence (WPPSI, and in their revised versions including the WISC-III and WPPSI-R) among feminine boys (Zucker & Bradley, 1995). This could point to an early developmental association between childhood gender nonconformity levels and later spatial cognition. Future studies using prospective designs may be able to test for any truly developmental associations. Among women, the association between CGN and time spent in the platform quadrant during the hidden trials was not in the expected direction (more feminine scores associated with more time in the quadrant). This is puzzling but given the lack of any predicted group differences between heterosexual and lesbian/bisexual women in the main outcomes, this finding should be viewed with caution.

Among men, the use of a landmark strategy was associated with more time in the platform quadrant, and the use of enfilading with longer search latencies but less time spent in the platform quadrant during hidden trials. It may be that men were using extra-maze cues (landmarks) to help remain in the correct quadrant which increased the time spent there. The use of enfilading (which involves making zigzag movements during navigation) would necessarily result in longer latencies and perhaps less time in the platform quadrant because it is a less efficient strategy for resolving the spatial location of a target. Among women, the use of landmark strategy was associated with shorter search latencies and more time in the platform quadrant during hidden trials. The use of a landmark strategy among women may improve their search latency and dwelling time because they rely more on extra-maze cues in the maze. In contrast to the mean group differences, these within-group analyses suggest that usage of landmarks is associated with improved spatial performance, albeit in specific components, among women (Dabbs et al., 1998; Saucier et al., 2002). For both sexes, the use of visual scanning compared to any other strategy was strongly associated with probe trial performance. This indicates, tentatively, that visual scanning-type strategies are important in the formation of spatial memory. It is important to remember that these results are limited by the fact that the initial run of the regression models suffered from high levels of multicollinearity and indicated a strong association between visual scanning and the main spatial measures. Thus, the results above reflect the predictive power of independent variables (such as CGN) on the variance left over after visual scanning was removed as a variable.

Several limitations of the present study merit comment. As we did not directly measure neural correlates, the present findings are silent on the neurobiology of the differences observed. Different patterns of spatial exploration responses, during the spatial learning phase of our task, may have contributed to the formation of an allocentric cognitive map used to promote later spatial recall (O’Keefe & Nadel, 1978). The hippocampus has long been proposed to have a critical role in the formation of allocentric spatial memory (e.g., Kessels et al., 2001), but evidence for structural and functional sex differences in humans is mixed (e.g., Blanch et al., 2004; Janzen & Van Turennout, 2004; cf. Ruigrok et al., 2014; see also Li et al., 2014). Nevertheless, performance on MWM tasks has been associated with hippocampal integrity, for example in patients with temporal lobe lesions (Astur et al., 2002; Parslow et al., 2005). Thus, future studies of sexual orientation-related differences in hippocampal structure and function are warranted. Limited neuroimaging studies suggest that volumetric patterns of brain asymmetry are more similar between gay men and heterosexual women and between heterosexual men and lesbian women, supporting the cross-sex hypothesis (Savic & Lindstrom, 2008). The present findings suggest that such neuroimaging studies could benefit from exploring the full range of variation in spatial performance between the groups (e.g., comparing subgroups with high and low scores as well as groups who use one strategy more than another). The present design may have been less sensitive to these more subtle individual differences which might be better indexed using imaging techniques.

It is possible that the use of spatial strategies is unrelated to hippocampal function but rather due to factors such as attention processes. We did not vary the potential size of the cognitive map formed during our task. In addition, attentional modulation by extra-maze cues may have affected any allocentric task processing. Future studies should aim to test several mazes varying in number of extra-maze cues to test whether sexual differences are associated with size of the cognitive map. Furthermore, the finding that heterosexual men were faster on the cue navigation trials suggest that there may be some basic visuomotor differences which require further investigation. Another intriguing possibility, while speculative, is that differences in approaching the task (e.g., that heterosexual men started faster and maintained this advantage throughout spatial learning) may reflect differences in personality traits such as risk-taking or extraversion. This requires further exploration in future work.

The results from the adjusted models also indicate that our sample was underpowered to detect some effects. While we did power our study for the primary outcomes, we did not do so for the covariates. Thus, power limitations precluded a satisfactory examination of the impact of age and IQ on our group differences. In general, the recruitment of sexual minority groups from community samples has the potential for bias although it is recognized in the field that random or representative sampling of this small and hard-to-reach population is difficult (Kuyper, Fernee, & Keuzenkamp, 2016). However, as sampling biases may apply more to our mean group differences than the within-group analyses future studies may benefit from methods such as targeted sampling. These would involve recruiting gay and lesbian participants from the same sources as heterosexual men and women and matching them for demographic variables.

In summary, the present findings, if replicated in larger samples, suggest that there are sexual orientation-related differences in spatial learning, spatial memory, and the spatial strategies used by humans on a commonly used measure of spatial memory. Further work is now needed to quantify how robust such differences are and whether they are associated with structural and/or functional differences in hippocampal regions of the brain.

## Footnotes

1 As this study focused on group effects, the results for block effects will not be reported here but can be provided upon request. In general, significant main effects of block (before adjustment for the covariates) were found on spatial learning and cued navigation measures, indicating participant improved in their performance across blocks. In spatial strategy usage during spatial learning, participants used more of each class of strategy across blocks.
